# Supplementary figures and images for: Phylogenomics resolves major relationships and reveals significant diversification rate shifts in the evolution of silk moths and relatives
Source: BMC Evol Biol. 2019 Sep 18;19:182. doi: 10.1186/s12862-019-1505-1 (PMC6751749; doi:10.1186/s12862-019-1505-1)

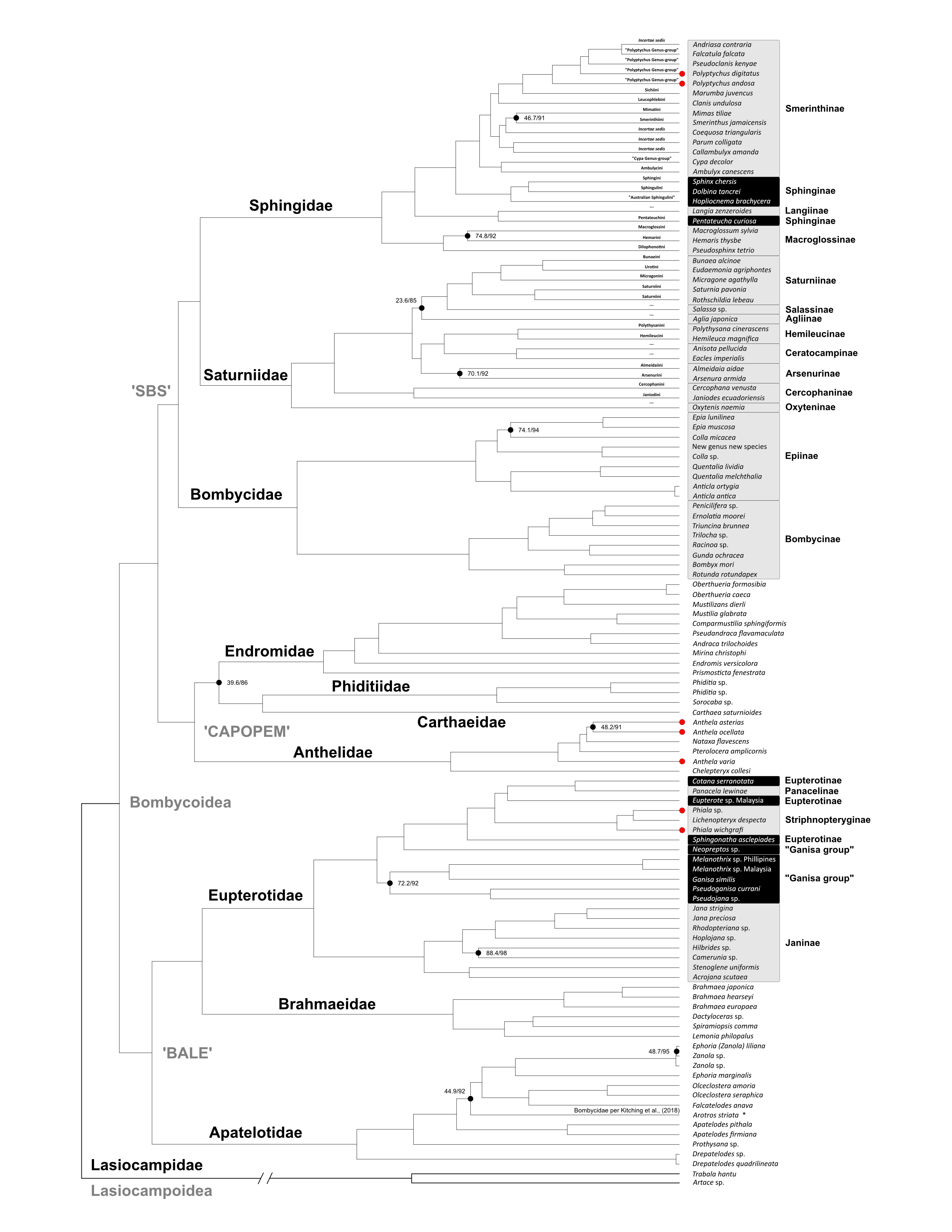

Supplement: Supplementary file 3 — Figure S1. Maximum likelihood tree of Bombycoidea, based on 650 AHE loci. All nodes are supported by ≥95% bootstrap values unless otherwise noted. Major taxonomic groups such as families, subfamilies, and tribes are labeled. Red circles at the tips correspond to genera that are not monophyletic. Black boxes around the tips correspond to non-monophyletic subfamilies. (TIFF 1188 kb) [file 12862_2019_1505_MOESM3_ESM.tiff]

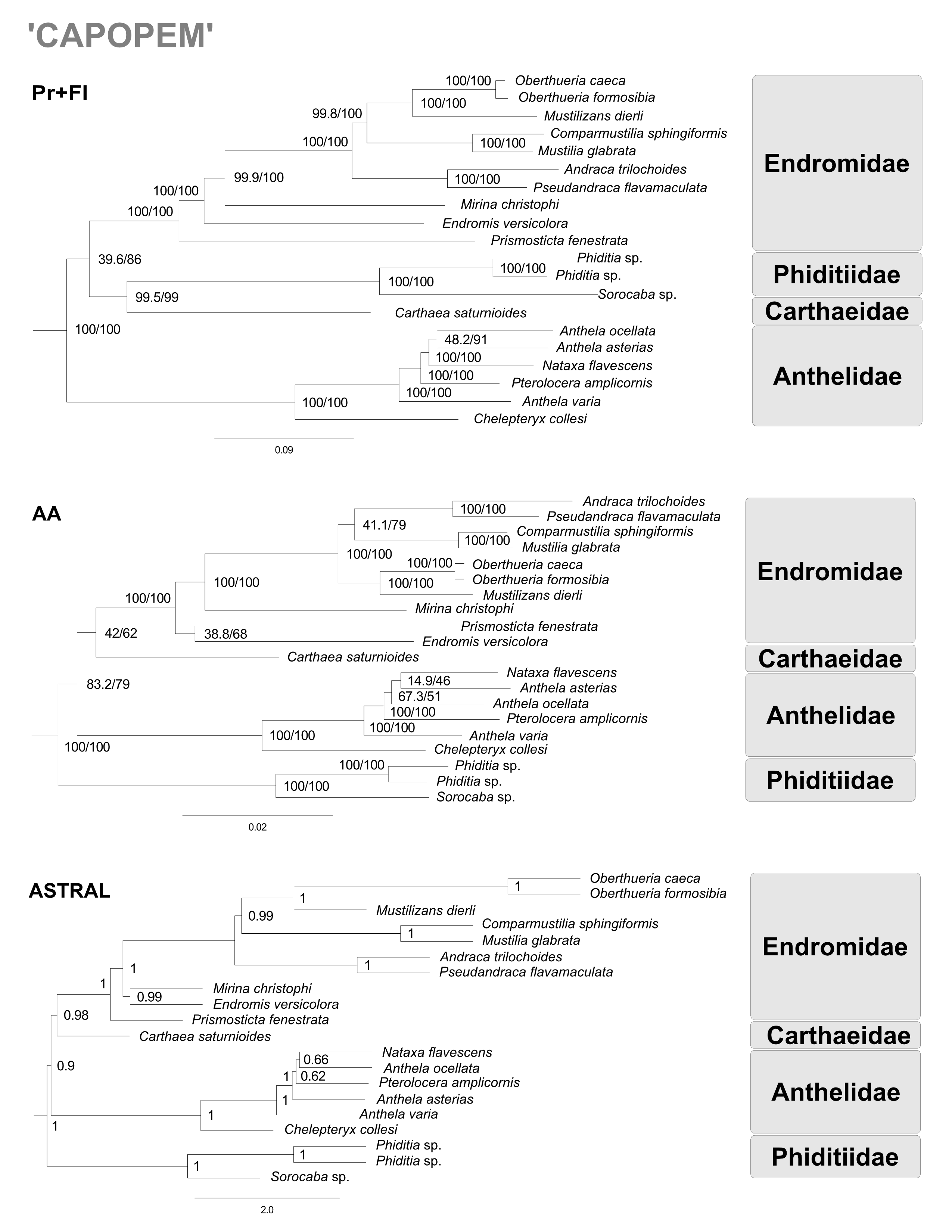

Supplement: Supplementary file 4 — Figure S2. Interfamilial relationships of the ‘CAPOPEM’ group. These relationships change depending on the data used (Pr + Fl or AA) to infer the phylogeny and the phylogenetic inference (supermatrix or ASTRAL). Values at the nodes of the Pr + Fl or AA trees indicate SH-aLRT/UFBS support, or ASV for the ASTRAL analysis. (TIFF 1294 kb) [file 12862_2019_1505_MOESM4_ESM.tiff]

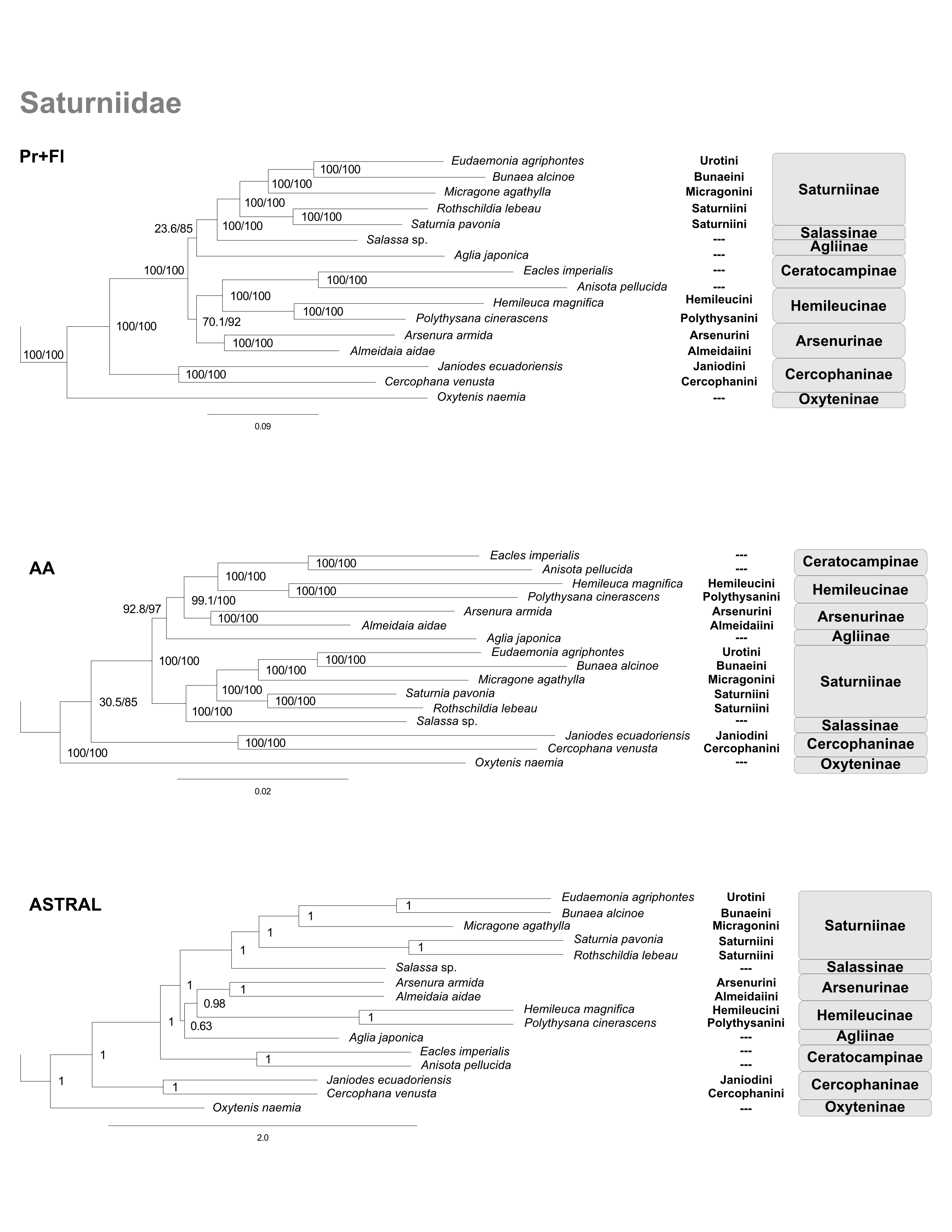

Supplement: Supplementary file 5 — Figure S3. Interfamilial relationships of the Saturniidae. These relationships change depending on the data used (Pr + Fl or AA) to infer the phylogeny and the phylogenetic inference method (supermatrix or ASTRAL). Values at the nodes of the Pr + Fl or AA trees indicate SH-aLRT/UFBS support, or ASV for the ASTRAL analysis. (TIFF 1224 kb) [file 12862_2019_1505_MOESM5_ESM.tiff]

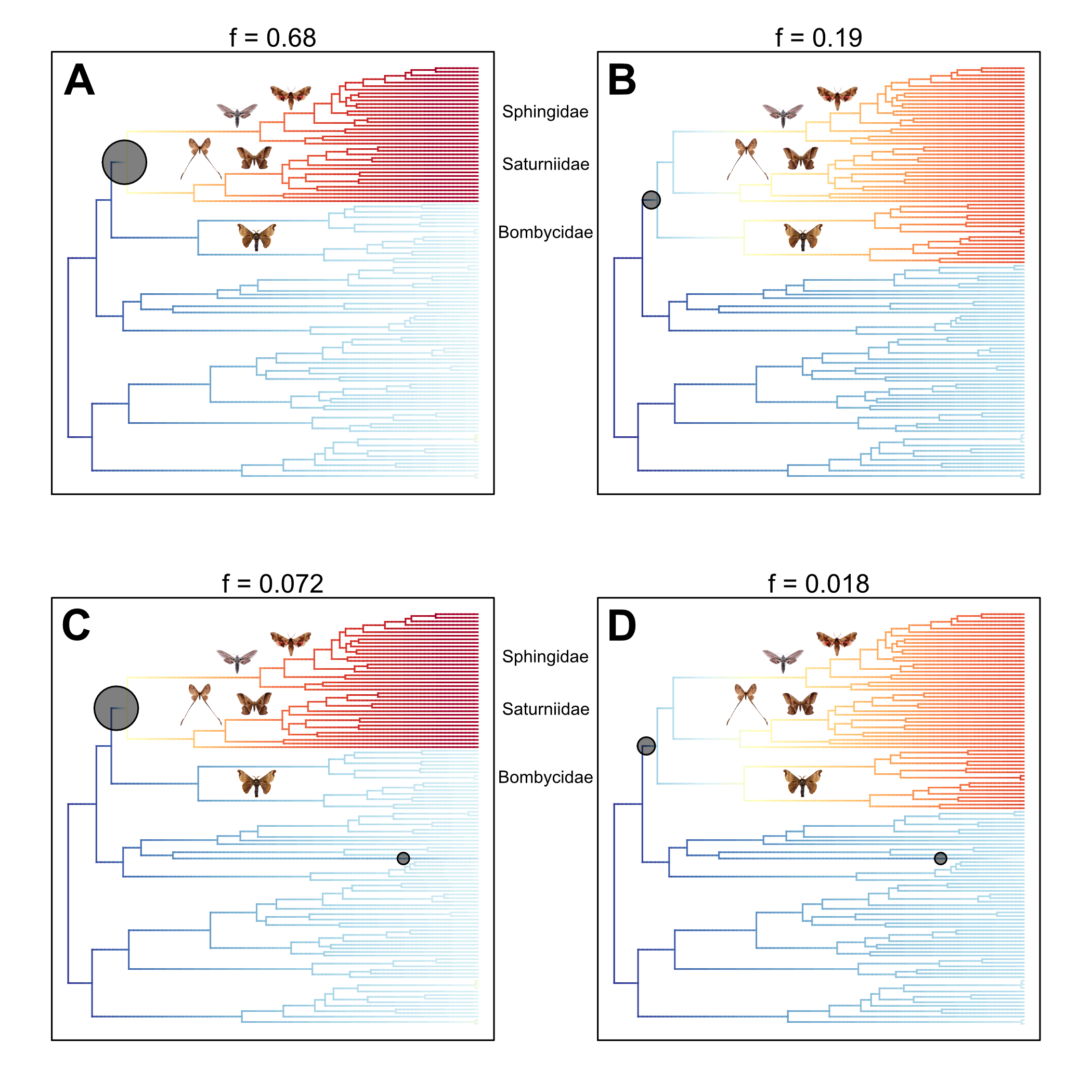

Supplement: Supplementary file 6 — Figure S4. The four most common 95% credible set of rate shift configurations sampled with BAMM. Branch color indicates the estimated diversification rate, with warmer colors representing lineages with higher rates. Major taxonomic groups with shifts are labeled. Photographs correspond to the major lineages (i.e., the SBS group) with diversification shifts in the phylogeny. (TIFF 1055 kb) [file 12862_2019_1505_MOESM6_ESM.tiff]
